# Childbearing May Increase the Risk of Nondiabetic Cataract in Chinese Women's Old Age

**DOI:** 10.1155/2015/385815

**Published:** 2015-08-16

**Authors:** Manqiong Yuan, Yaofeng Han, Ya Fang, Cheng-I Chu

**Affiliations:** ^1^State Key Laboratory of Molecular Vaccinology and Molecular Diagnostics, School of Public Health, Xiamen University, Xiamen 361102, China; ^2^Key Laboratory of Health Technology Assessment in Fujian Province University, School of Public Health, Xiamen University, Xiamen 361102, China; ^3^Department of Public Health, Tzu Chi University, Hualien 97004, Taiwan

## Abstract

*Backgrounds*. Ocular changes may arise during pregnancy and after childbirth, but very few studies have reported the association between childbearing and cataract among older adults.* Methods*. 14,292 individuals aged 60+ years were recruited in Xiamen, China, in 2013. Physician-diagnosed cataract and diabetes status were assessed by a self-reported questionnaire. Childbearing status was measured by number of children (NOC). Structural equation modeling (SEM) analysis was conducted to examine the relationships among NOC, diabetes, and cataract. Gender-specific logistic models regressing nondiabetic cataract on NOC were performed by adjusting some covariates.* Results*. 14,119 participants had complete data, of whom 5.01% suffered from cataract, with higher prevalence in women than men (6.41% versus 3.51%). Estimates of SEM models for women suggested that both NOC and diabetes were risk factors for cataract and that no correlation existed between NOC and diabetes. Women who had one or more children faced roughly 2–4 times higher risk of nondiabetic cataract than their childless counterparts (OR [95% CI] = 3.88 [1.24, 17.71], 3.21 [1.04, 14.52], 4.32 [1.42, 19.44], 4.41 [1.46, 19.74], and 3.98 [1.28, 18.10] for having 1, 2, 3, 4-5, and 6 or more children, resp.).* Conclusions*. Childbearing may increase the risk of nondiabetic cataract in Chinese women's older age.

## 1. Introduction

According to the two latest WHO assessments, cataract has consistently been the leading cause of world blindness, which was responsible for around 48% and 51% of blindness in 2002 [1] and 2010 [2], respectively. Biological ageing plays the most crucial role in the development of cataract [3–5]. China, the most populous country, has been experiencing an unprecedented ageing due to a lower birth rate [6] and longer life expectancy [7]. About 15.5% of the entire Chinese were aged 60+ years in 2014, a figure much higher than the threshold for ageing society (10%). Therefore as an age-related disease, cataract requires increased and urgent attention in China [8, 9].

Besides age, many other related factors, such as diabetes and gender, have also been well-addressed in previous studies [10–13]. It has been demonstrated that diabetics faced 2–5 times greater risk of developing cataracts than the nondiabetic counterparts [12]. Additionally, gender diversity in the prevalence of cataract has also been frequently reported and most studies showed greater prevalence among females than males [11]. Hormonal differences between men and women may mainly contribute to such gender diversity [10, 14], but the reasons were still not fully understood.

Ocular changes may arise during pregnancy due to the modifications of hormone, metabolism, and weight [15]. Although most of the changes are reversible, some are occasionally permanent which may in turn cumulatively affect women's vision at their older ages [16]. A study revealed that there was a significant association between parity and the risk of cataract among middle- and older age women [17]. However, the evidence is scant since very few studies have directly reported the link between childbearing and cataract, especially for women in their old ages. In this study, we aim to (1) test the relationship between childbearing and cataract among the older women and (2) examine whether diabetes is a mediator in this relationship.

## 2. Methods

### 2.1. Study Population

As previously described [18], we conducted a large scale cross-sectional survey among 14,292 older adults aged 60+ years in Xiamen, China, in 2013. The participants were enrolled by a multistage sampling procedure. In the first stage, all 38 subdistricts in Xiamen were selected. In stage 2, one-third of communities were randomly sampled from each subdistrict and a total of 173 communities were included in the end. The randomization of these communities was performed by computer-generated random numbers. In stage 3, participants were conveniently selected from each community by controlling for gender and age composition. The number of individuals to be sampled in each community was determined according to its proportion of eligible older adults. Participants' demographic characteristics, activities of daily living, physical health, psychological health, and social support were assessed by a structural questionnaire, which was finished by a face-to-face interview. Written informed consent was obtained by each participant and our study was approved by the ethical review committee of School of Public Health, Xiamen University.

### 2.2. Measurements

The primary outcomes in this study were whether the participants suffered from physician-diagnosed cataract and diabetes. They were assessed by the same item: “Do you suffer from the following physician-diagnosed chronic diseases? (check all that apply).” Cataract and diabetes were two of the fifteen listed chronic diseases. Only if the option was ticked, we assume the participant suffered from the corresponding chronic disease. In Xiamen, for medical screening purposes, people who were aged 60 years or older can participate in an annual physical examination for free in recent years, including blood pressure and blood glucose checks, and an ocular examination. Moreover, some self-reported chronic diseases were reported to be highly correlated with physician's records [19]. Therefore, the reliability of self-reported diabetes and cataract in the elderly should be potentially ensured (see Supplementary 1 for the reason in detail in Supplementary Material available online at http://dx.doi.org/10.1155/2015/385815). The nondiabetic cataracts in our study referred to the individuals who suffered from cataract but did not have diabetes. The exposure of interest was childbearing status which was measured by number of children (NOC). It was assessed by the item of “How many children have you had?” Numerical response was obtained and was classified into six levels: 0, 1, 2, 3, 4-5, and 6 or more. Additionally, some basic information (gender, age, residence, education, and marital status), life habits (dietary salt intake, smoking history, and alcohol drinking), and hypertension status were included as covariates (see Table 1 for the details of the classification of the variables). We considered the hypertension status as a covariate because it has also been frequently reported to have relationship to cataract [20].

### 2.3. Analytical Strategies

First, we summarized the characteristics of participants by cataract status using descriptive statistics (mean and standard deviation for age and counts and proportions for the other categorical characteristics). Chi-square tests were performed to assess the relationship between cataract and all the other covariates. Second, to identify the relationships among NOC, diabetes, and cataract in women, Stata v 13.0 was used to perform structural equation modeling (SEM). The reason we tested their relationships was due to the concern that diabetes may be a mediator in the relationship between NOC and cataract. The SEM for this mediation model for the *i*th participant (1 ≤ *i* ≤ *n*) is given by

(1) logitProbabilityDi=β0+β1NOCi+εi,logitProbabilityCi=γ0+γ1Di+γ2NOCi+γ3TCovariates+δi,

where the outcome *D*_*i*_ is diabetic status, *C*_*i*_ is cataract status, *β*_0_, *β*_1_, *γ*_0_, *γ*_1_, *γ*_2_, and **γ**_3_^**T**^ are the regression coefficients,* Covariates* are the nine covariates described above, and *ε*_*i*_ and *δ*_*i*_ are the random errors. We assume that the error terms (*ε*_*i*_, *δ*_*i*_) are uncorrelated. Third, we presented the prevalence of cataract by line chart under the six levels of NOC, stratified by gender and diabetes status. Fourth, logistic regressions were carried out to model the cataract status on the NOC among the participants without diabetes, stratified by gender and adjusted by the nine covariates mentioned above. *P* < 0.05 indicated that the associations were statistically significant in our study.

## 3. Results

Among the 14,292 participants, 14,119 had complete data on all variables mentioned above and were included in the following analyses. Descriptive statistics of characteristics stratified by gender and cataract status were presented in Table 1. Among 14,119 valid participants, the prevalence of cataract was 5.01%, with an obviously higher prevalence in women than men (6.41% versus 3.51%). The average age was significantly higher in cataract participants than in those without cataract for both men and women (*P* < 0.001). An increase trend of prevalence of cataract was presented as NOC grew for women and ranged from 3.10% for the childless to 9.09% for those who had six or more children. However, such trend disappeared in men. About ten percent of participants suffered from diabetes, of whom the prevalence values of cataract were 4.48% for men and 9.26% for women, which were both higher than their nondiabetic counterparts. Approximately thirty percent of participants reported to have hypertension and 6.85% of them reported to have cataract. Significantly higher prevalence of cataract was presented in hypertensive participants than nonhypertensive groups for both men and women. Female participants who had salt-heavy diet, were illiterate, were single or widowed, and had quit drinking had significantly higher prevalence of cataract (*P* < 0.05). However, for male participants, except for the hypertensive status, only smoking history was significantly associated with cataract status. Unexpectedly, men who had quit smoking presented highest prevalence, and those who smoked often showed lowest one.

**Figure 1:**
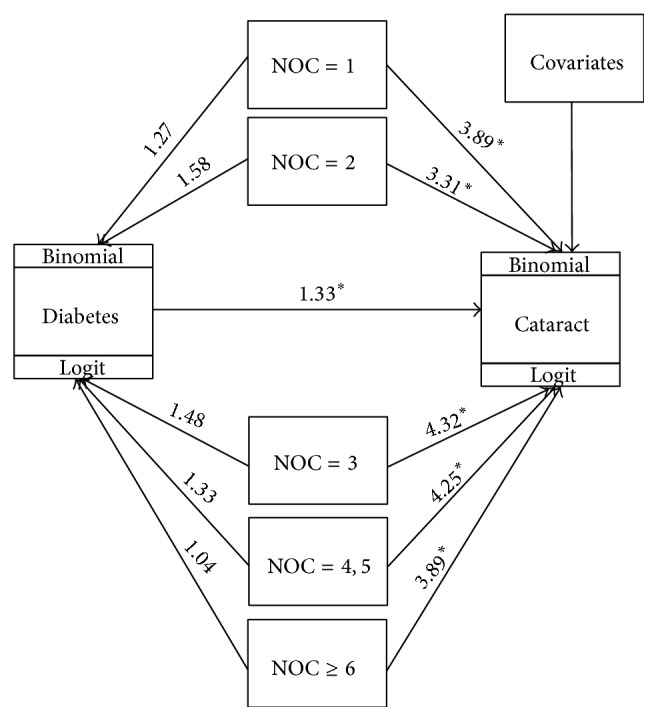
Path diagram of structural equation modeling to depict the relationships among number of children (NOC), diabetes, cataract, and the other nine covariates. The covariates included age, hypertension status, dietary salt intake, residence, education, occupation, marital status, smoking history, and alcohol drinking (^*∗*^ *P* < 0.05).

As descriptive results shown above, both diabetes and having more children increase the risk of cataract for older women. To test whether diabetes is a mediator in the relationship between NOC and cataract, mediation analysis with SEM was performed.  Figure 1 presented the path diagram with estimated odds ratios (OR), which represented the SEM models for female elderly (equation (1)). Diabetic women had a statistically higher risk (OR = 1.33) of cataract than their nondiabetic counterparts. Those who had one or more children faced roughly 2–4 times higher risk of cataract than the childless women. However, all the paths between NOC and diabetes were not significant, indicating that diabetes should not be a mediator in the relationship between NOC and cataract. The details of the estimates of the SEM models can be found in Supplementary 2.

**Figure 2:**
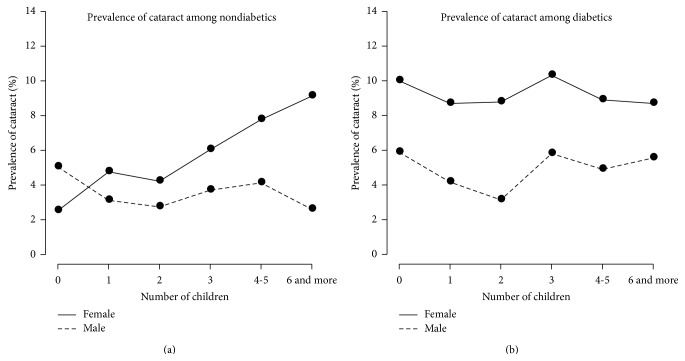
Line charts of the prevalence of cataract under the six levels of number of children, stratified by gender and diabetes status. The left panel was the prevalence of cataract among the 12,749 nondiabetic participants while the right one was among 1,370 diabetic participants.

The prevalence of cataract under the six levels of NOC stratified by gender and diabetic status was depicted by line chart in Figure 2. The left panel was the prevalence of cataract among the 12,749 nondiabetic participants. A notably increased prevalence (solid line) appeared as NOC grew for nondiabetic older women, but it disappeared among the nondiabetic older men (dotted line). Moreover, the nondiabetic females had higher prevalence of cataract than nondiabetic males at all levels of NOC except among the childless participants. The right panel was the prevalence of cataract among 1,370 diabetic participants. At all the levels of NOC, diabetic females had obvious higher prevalence of cataract than diabetic males. The increased trend of prevalence as having more children disappeared for diabetic female and male participants.

Table 2 presented the ORs with corresponding 95% confidence intervals (CIs) obtained from logistic regression models for nondiabetic participants stratified by gender. For both nondiabetic women and men, age was a crucial risk factor for developing cataract. The older women who had one or more children faced roughly 2–4 times higher risk of nondiabetic cataract than their childless counterparts (OR [95% CI] = 3.88 [1.24, 17.71], 3.21 [1.04, 14.52], 4.32 [1.42, 19.44], 4.41 [1.46, 19.74], and 3.98 [1.28, 18.10] for having 1, 2, 3, 4-5, and 6 or more children, resp.). However, among the nondiabetic older men, the ORs seemed to decrease as NOC grew, although they were not statistically significant. Hypertension, salt-heavy diet, and living in a city were risk factors for women but not for men. Unexpectedly, having quit smoking was a risk factor, and being widowed was a protect factor for men only.

## 4. Discussion

The findings in this study indicated that childbearing may increase the risk of nondiabetic cataract in women's old age. After controlling for the potential confounders, nondiabetic women who had one or more children faced roughly 2–4 times higher risk of cataract than the childless. Moreover, SEM analysis suggested that diabetes was not a mediator in the relationship between NOC and cataracts. These results may contribute to the existing body of literature on identifying the risk factor of cataract among the older individuals.

The pathomechanism underlying the association between childbearing and cataract remains unclear. However, some possible explanations for the childbearing effect in older women may be speculated; these include (1) pregnancy-induced estrogen changes [21]. Some investigators believed that estrogen played a protective role in controlling the development of cataract [10]. A previous study indicated that the risk of cortical cataract increased by about 5% for each one year increment in age at menarche and that the risk decreased by 11% for each five years of age older at menopause [22]. Therefore, we may postulate that the increased risk of cataract for women having more children is due to a reduction in lifetime exposure to circulating estrogens induced by each pregnancy and breastfeeding. (2) Postpartum obesity may be another important risk factor of cataract for women in their later life [23, 24]. A previous study presented that the average gestational weight gains for Chinese women (17.3 ± 4.9 kg, 17.4 ± 4.4 kg, and 15.7 ± 5.1 kg for women of low, moderate, and high prepregnancy body mass index, resp.) [25] were much higher than the recommended criteria [26] (13–16.7 kg, 11–16.4 kg, and 7.1–14.4 kg, resp.). (3) Various ocular changes occur during pregnancy, including decreased sensitivity of the cornea, enlarged blind spots, and bitemporal loss [27, 28]. The cumulative effect of those asymptomatic and subtle ocular changes may also contribute to the development of cataract for women in later life. (4) A complex of other risk factors, such as the stress for taking care of many children, may also increase the risk of cataract. However, in either case, more physiological studies were in great need to fully elucidate the mechanism.

In line with many previous studies [29–31], cataract was more common in women than men, with the prevalence of 6.41% versus 3.51% in the present study. Such difference is often addressed by the estrogen deficiency [10] or biomass cooking fuels [32]. However, this explanation could not fully elucidate our results. In the present study the prevalence of cataract among childless women was notably lower than that among childless men (3.1% versus 5.1%). This result may further indicate that childbearing was an important risk factor of cataract among older women. A study has directly stated that the risk of cataract in young women (35–45 years) increased by an estimated 20% for each additional birth [33]. Moreover, older women (45–86 years) were observed to have 11.3% higher risk of cataract for each additional live birth in a recent cohort study [17]. Among the nondiabetic women, the prevalence of cataract grew as they had more children, but such trend disappeared among the women with diabetes. In other words, the effect of childbearing on cataract among the female older adults may be mediated or covered by the effect of diabetes. Cataract was the second most ocular common complication of diabetes [34], and the effect of diabetes on cataract should be direct and obvious. However, the childbearing occurred at women's younger age and its effect on age-related cataract may be cumulative and subtle. Therefore the effect of childbearing on cataract is easy to be neglected or covered by other stronger risk factors.

Consistent with many previous studies [35–37], our findings identified hypertension, salt-heavy diet, living in a city, and having quit smoking as risk factors for development of cataract. Salt-heavy diet has been well-addressed to be related to hypertension [38, 39], which may increase the risk of development of the posterior subcapsular cataracts [40, 41]. As for smoking, it had been consistently stated to be associated with both nuclear and posterior subcapsular cataract [35]. In this study, having quit smoking was a risk factor for older men which might be due to the smoking history in their younger age. Some investigators found that risk of cataract remains elevated for many years following smoking cessation [42]. Unexpectedly, we found men who smoked often had obviously lower prevalence of cataract than those who never smoked (2.81% versus 3.61%). Such paradoxical result has also been presented in other studies [43]. To find the reason, we further analyzed our data and found that individuals who smoked often were significantly younger than those who never smoked (69.45 years versus 71.78 years, *P* < 0.01), especially among men (69.12 years versus 71.77 years, *P* < 0.01). Therefore, age, the most critical risk factor of cataract, probably confounded the relationship between cataract and smoking history. This speculation was further validated in the following regression model for men where OR for smoking often was higher than never smoking (1.07 versus 1.00), after controlling for age and other impact factors. Another possible explanation is that some related chronic diseases can prompt smoking cessation for older adults, and the current smokers may have or believe they have relatively better physician conditions. However, this speculation cannot be validated in this study due to our cross-sectional survey and limited measurements. To fully uncover the relationship between smoking and cataract, variables such as smoking pack-years, age of smoking cessation, and the reason(s) for smoking cessation are in great need. Despite the fact that abovementioned risk factors of cataracts were well-demonstrated, limited studies have elucidated the gender-specified risk factors. Our results suggested that salt-heavy diet and hypertension were the risk factors for nondiabetic female only and on the contrary, having quit smoking was for nondiabetic male only. More precise study design and analyses were needed to explore such gender disparities.

The findings in the present study have uncovered the relationship between childbearing and cataract in older adults. They may open new research challenges to detect the risk factors of senile cataract. Nevertheless, some limitations should be acknowledged. First, the chronic conditions were obtained by a self-reported question. As a result, we may have underestimated the true prevalence of cataract and diabetes. However, substantial agreement was found in a study comparing subjects' self-reported diabetes with information from medical records [19]. Some studies demonstrated that self-reported diabetes was >92% reliable over time [44] and it was a reliable proxy for medical record review [45]. The difference between self-reporting and true prevalence of cataract has been evaluated in Supplementary 1, and the reliability of self-reported cataract in this study may also be potentially ensured. Second, to exclude the diabetes-induced cataracts, the data of the participants with diabetes were all removed from our regression models. However, some of participants may have cataract prior to diabetes or the cataracts were not diabetes-induced, and as a result, the complete exclusion may consequently result in some bias. Nevertheless, such bias was restricted, since only 71 (less than 1%) female participants had both cataract and diabetes. Third, there are three primary types of age-related cataracts (nuclear, cortical, and posterior subcapsular), and each may have its own somewhat varying causes [37, 41]. However, the types of cataracts were not asked in our survey and therefore we cannot specify which type(s) of cataract were accounted for in relation to childbearing.

## 5. Conclusions

This study has revealed a relationship between childbearing status and the risk of cataract among Chinese elderly, and diabetes was not a mediator in this relationship. As a result, we should pay more attention to the eyes care for the women during pregnancy and after childbirth.

## Supplementary Material

(1)Description for Supplementary 1: Some comments of the difference between the self-reporting and true prevalence of diabetes/cataract. Although the prevalence of diabetes/cataract should be underestimated when using self-reported data, their reliabilities in this study may be potentially ensured.
(2)Description for Supplementary 2: Estimates of structural equation modeling (SEM) stratified by gender. Due to the concern that diabetes may be a mediator in the relationship between childbearing and cataract, we tested their relationships using SEM models.

## Figures and Tables

**Table 1:** Summary of gender stratified basic characteristics of 14,119 participants.

Characteristic	Male (*N* = 6,806)				Female (*N* = 7,313)			
	Noncataract	Cataract	^a^Prevalence (%)	^b^ *P*	Noncataract	Cataract	^a^Prevalence (%)	^b^ *P*
Total, *N*	6567	239	3.51		6844	469	6.41	
Age, mean (SD)/years	70.81 (7.73)	75.00 (7.42)		<0.001	71.75 (8.78)	75.75 (8.49)		<0.001
NOC, *N* (%)				0.141				<0.001
0	242 (3.69)	13 (5.44)	5.10		125 (1.83)	4 (0.85)	3.10	
1	1110 (16.90)	37 (15.48)	3.23		904 (13.21)	49 (10.45)	5.14	
2	1924 (29.30)	55 (23.01)	2.78		1662 (24.28)	83 (17.70)	4.76	
3	1706 (25.98)	69 (28.87)	3.89		1880 (27.47)	131 (27.93)	6.51	
4 or 5	1306 (19.89)	57 (23.85)	4.18		1753 (25.61)	150 (31.98)	7.88	
6 or more	279 (4.25)	8 (3.35)	2.79		520 (7.60)	52 (11.09)	9.09	
Diabetes status, *N* (%)				0.177				<0.001
Nondiabetic	5991 (91.23)	212 (88.70)	3.42		6148 (89.83)	398 (84.86)	6.08	
Diabetic	576 (8.77)	27 (11.30)	4.48		696 (10.17)	71 (15.14)	9.26	
Hypertension status, *N* (%)				0.012				<0.001
Nonhypertensive	4668 (71.08)	152 (63.60)	3.15		4785 (69.92)	265 (56.50)	5.25	
Hypertensive	1899 (28.92)	87 (36.40)	4.38		2059 (30.08)	204 (43.50)	9.01	
Dietary salt intake, *N* (%)				0.975				0.006
Salt-light (<6 g/day)	2618 (39.87)	97 (40.59)	3.57		3463 (50.6)	233 (49.68)	6.30	
Salt-medium (6–18 g/day)	3313 (50.45)	120 (50.21)	3.50		2975 (43.47)	191 (40.72)	6.03	
Salt-heavy (≥18 g/day)	636 (9.68)	22 (9.21)	3.34		406 (5.93)	45 (9.59)	9.98	
Residence, *N* (%)				0.159				0.532
City	3158 (48.09)	126 (52.72)	3.84		3371 (49.25)	238 (50.75)	6.59	
Rural	3409 (51.91)	113 (47.28)	3.21		3473 (50.75)	231 (49.25)	6.24	
Education, *N* (%)				0.146				<0.001
Illiterate	1103 (16.8)	42 (17.57)	3.67		3223 (47.09)	267 (56.93)	7.65	
Primary	2348 (35.75)	88 (36.82)	3.61		1824 (26.65)	119 (25.37)	6.12	
Junior high school	1634 (24.88)	45 (18.83)	2.68		952 (13.91)	35 (7.46)	3.55	
Senior high school and beyond	1482 (22.57)	64 (26.78)	4.14		845 (12.35)	48 (10.23)	5.38	
Occupation, *N* (%)				0.485				0.395
Employed	2059 (31.35)	86 (35.98)	4.01		1441 (21.05)	88 (18.76)	5.76	
Farmer	2905 (44.24)	101 (42.26)	3.36		2764 (40.39)	195 (41.58)	6.59	
Jobless	331 (5.04)	11 (4.60)	3.22		1337 (19.54)	103 (21.96)	7.15	
Others	1272 (19.37)	41 (17.15)	3.12		1302 (19.02)	83 (17.70)	5.99	
Marital status, *N* (%)				0.433				<0.001
Inmarriage	5575 (84.89)	200 (83.68)	3.46		3839 (56.09)	208 (44.35)	5.14	
Single	147 (2.24)	9 (3.77)	5.77		31 (0.45)	3 (0.64)	8.82	
Divorced	81 (1.23)	2 (0.84)	2.41		76 (1.11)	3 (0.64)	3.80	
Widowed	764 (11.63)	28 (11.72)	3.54		2898 (42.34)	255 (54.37)	8.09	
Smoking history, *N* (%)				<0.001				0.293
Never	2139 (32.57)	80 (33.47)	3.61		6340 (92.64)	437 (93.18)	6.45	
Sometimes	1558 (23.72)	48 (20.08)	2.99		300 (4.38)	15 (3.20)	4.76	
Often	2113 (32.18)	61 (25.52)	2.81		137 (2.00)	9 (1.92)	6.16	
Quit	757 (11.53)	50 (20.92)	6.20		67 (0.98)	8 (1.71)	10.67	
Alcohol Drinking, *N* (%)				0.235				0.025
Never	2873 (43.75)	106 (44.35)	3.56		6141 (89.73)	423 (90.19)	6.44	
Sometimes	2544 (38.74)	87 (36.40)	3.31		607 (8.87)	33 (7.04)	5.16	
Often	654 (9.96)	20 (8.37)	2.97		45 (0.66)	4 (0.85)	8.16	
Quit	496 (7.55)	26 (10.88)	4.98		51 (0.75)	9 (1.92)	15.00	

^a^Prevalence of cataract.

^b^ *P* value of chi-square test to assess the relationship between cataract and the other variables.

NOC: number of children; BMI: body mass index.

**Table 2:** Odds ratio (OR) with 95% confidence intervals (CIs) of gender-specific logistic regressions for nondiabetic datasets.

Characteristic (reference)	Male (*N* = 6,203)		Female (*N* = 6,546)	
	OR	95% CI	OR	95% CI
Age	1.08^*∗∗∗*^	1.06–1.11	1.04^*∗∗∗*^	1.03–1.06
NOC (0)				
1	0.95	0.39–2.57	3.88^*∗*^	1.24–17.71
2	0.69	0.29–1.83	3.21^*∗*^	1.04–14.52
3	0.77	0.33–2.02	4.32^*∗*^	1.42–19.44
4 or 5	0.67	0.28–1.77	4.41^*∗*^	1.46–19.74
6 or more	0.30^*∗*^	0.09–0.95	3.98^*∗*^	1.28–18.10
Hypertension status (nonhypertensive)				
Hypertensive	1.24	0.92–1.67	1.68^*∗∗∗*^	1.35–2.08
Dietary salt intake (salt-light (<6 g/day))				
Salt-medium (6–18 g/day)	1.15	0.85–1.56	1.07	0.86–1.33
Salt-heavy (≥18 g/day)	0.96	0.56–1.60	1.68^*∗∗*^	1.12–2.46
Residence (city)				
Rural	0.91	0.55–1.49	0.73^*∗*^	0.53–1.00
Education (illiterate)				
Primary	1.35	0.90–2.07	0.91	0.68–1.2
Junior high school	1.18	0.70–1.98	0.46^*∗∗*^	0.27–0.75
Senior high school and beyond	1.50	0.86–2.64	0.69	0.42–1.11
Occupation (employed)				
Farmer	1.20	0.69–2.12	1.06	0.68–1.67
Jobless	1.10	0.50–2.25	1.02	0.68–1.54
Others	0.88	0.56–1.37	0.83	0.56–1.23
Marital status (inmarriage)				
Single	1.49	0.50–4.28	2.80	0.41–11.66
Divorced	0.75	0.12–2.53	0.88	0.21–2.46
Widowed	0.60^*∗*^	0.37–0.94	0.97	0.75–1.24
Smoking history (never)				
Sometimes	0.85	0.55–1.30	0.56^*∗*^	0.29–0.99
Often	1.07	0.71–1.62	0.64	0.26–1.34
Quit	1.92^*∗*^	1.21–3.02	1.01	0.35–2.46
Alcohol drinking (never)				
Sometimes	1.11	0.79–1.56	1.05	0.68–1.55
Often	1.22	0.70–2.06	1.55	0.44–4.17
Quit	0.94	0.53–1.60	1.61	0.50–4.36

^*∗*^ *P* value < 0.05, ^*∗∗*^ *P* value < 0.01, ^*∗∗∗*^ *P* value < 0.001.

NOC: number of children; BMI: body mass index.
