# Supplementary material for: Childbearing May Increase the Risk of Nondiabetic Cataract in Chinese Women's Old Age
Source: J Ophthalmol. 2015 Aug 16;2015:385815. doi: 10.1155/2015/385815 (PMC4553188; doi:10.1155/2015/385815)
Supplement: Supplementary file 1 — (1)Description for Supplementary 1: Some comments of the difference between the self-reporting and true prevalence of diabetes/cataract. Although the prevalence of diabetes/cataract should be underestimated when using self-reported data, their reliabilities in this study may be potentially ensured. (2)Description for Supplementary 2: Estimates of structural equation modeling (SEM) stratified by gender. Due to the concern that diabetes may be a mediator in the relationship between childbearing and cataract, we tested their relationships using SEM models. [file 385815.f1.pdf]

Supplementary 1

The difference between the self-reporting and true prevalence of diabetes/cataract

It is a major limitation of this study that the status of cataract and diabetes were self-reported. In general, the prevalence of diabetes/cataract should be underestimated when using self-reported data.

For self-reported diabetes, some studies demonstrated that it was>92% reliable over time[1] and it was a reliable proxy for medical record review[2]. Moreover, substantial agreement was found in a study by comparing subjects' self-reported diabetes with information from records[3]. Based on these, the reliability of self-reported diabetes in this study may be potentially ensured.

For cataract, the difference of self-reporting and true prevalence, as addressed by[4], may not be easy to compare because different studies have used different definitions, detection and grading techniques. Moreover, it may present a great diversity based on ethnicity and location, even when using the same examination protocol and cataract classification method. For example, a study found that the prevalence of cataract was 2.1% for white women aged 45-64 years, and it was 300% higher for black women at the same age level[5]. Another study found the prevalence of cataract in Tibet (a western province in China) was 60% higher than in Shunyi (a county in Beijing, China), 14.6% vs. 9.1% (age- and sex-adjusted

comparison)[6, 7]. Therefore, to estimate the difference between self-reported cataract prevalence and true prevalence in Xiamen older population, it is best to find some reports that have provided the local prevalence of cataract and the cataract as diagnosed by some standard clinical criteria. Unfortunately, no such studies were found till now. In light of this, we chose to use a national representative study. We found a cohort study involving 27, 009 eligible participant aged 45-86 years in China between 2008 and 2010, wherein all participants took the ocular examination using a slit lamp, and the Lens Opacity Classification System (LOCS II) was used for lens grading. They defined a definite cataract as LOCS II grade 2 or worse for at least one of the three main types of cataract, including nuclear, cortical and posterior subcapsular cataract[8]. The prevalence of cataract for the 14,337 women (excluding the childless women) was 6.78%. In this study, if we excluded the childless women, the prevalence of self-reported cataract was 6.47% in women (4.5% lower, which may be acceptable). However, the difference should be greater because the age range in that cohort study was younger than that in our study. Unfortunately, the prevalence of cataract under different age levels was not provided in their report, and thus we could not measure the prevalence difference directly. Additionally, within the past few years in Xiamen, for medical screening purposes, people who were aged 60 years or older can participate in an annual physical examination for free, including blood pressure and blood glucose checks, and an ocular examination. Therefore, it may be easier for older adults in Xiamen to be aware of their chronic diseases, including cataract.

Based on these, the reliability of self-reported cataract in this study may also be potentially ensured.

References

1. Schneider AL, Pankow JS, Heiss G, Selvin E. Validity and reliability of self-reported diabetes in the atherosclerosis risk in communities study. American journal of epidemiology. 2012;176(8):738-43. doi: 10.1093/aje/kws156. PubMed PMID: 23013620; PubMed Central PMCID: PMC3571247.

2. Goldman N, Lin IF, Weinstein M, Lin YH. Evaluating the quality of self-reports of hypertension and diabetes. Journal of clinical epidemiology. 2003;56(2):148-54. PubMed PMID: 12654409.

3. Okura Y, Urban LH, Mahoney DW, Jacobsen SJ, Rodeheffer RJ. Agreement between self-report questionnaires and medical record data was substantial for diabetes, hypertension, myocardial infarction and stroke but not for heart failure. Journal of clinical epidemiology. 2004;57(10):1096-103. doi: 10.1016/j.jclinepi.2004.04.005. PubMed PMID: 15528061.

4. Hammond C. The epidemiology of cataract 2001 [cited 2015 Jul 16]. Available from: http://www.optometry.co.uk/uploads/articles/10f51a3c1d3ebf7b0754b5d39e12bf4f_hammond20010209.pdf.

5. Klein BE, Klein R. Cataracts And Macular Degeneration In Older Americans. Arch Ophthalmol-Chic. 1982;100(4):571-3. PubMed PMID: WOS:A1982NL92900001.

6. Hu TS, Zhen Q, Sperduto RD, Zhao JL, Milton RC, Nakajima A. Age-related cataract in the Tibet Eye Study. Archives of ophthalmology. 1989;107(5):666-9. PubMed PMID: 2719577.

7. Zhou Q, Friedman DS, Lu H, Duan X, Liang Y, Yang X, et al. The epidemiology of age-related eye diseases in Mainland China. Ophthalmic epidemiology. 2007;14(6):399-407. doi: 10.1080/09286580701331974. PubMed PMID: 18161614.

8. Tian Y, Wu J, Xu G, Shen L, Yang S, Mandiwa C, et al. Parity and the risk of cataract: a cross-sectional analysis in the Dongfeng-Tongji cohort study. The British journal of ophthalmology. 2015. doi: 10.1136/bjophthalmol-2015-306653. PubMed PMID: 25995298.

Supplementary 2

Table S1. Estimates of structural equation modeling stratified by gender

	**Characteristic** (reference)	**Female (*N*=6,546)**		**Male (*N*=6,203)**	
		**OR**	**95% CI**	**OR**	**95% CI**
**Equation 1**	**NOC** (0)				
	1	1.27	0.64-2.51	1.64	0.97-2.77
	2	1.58	0.82-3.07	1.50	0.89-2.50
	3	1.48	0.77-2.87	1.34	0.80-2.25
	4 or 5	1.33	0.68-2.57	1.13	0.67-1.93
	6 or more	1.04	0.51-2.12	0.94	0.47-1.86
**Equation 2**	**Age**	1.04	1.03-1.06	1.08	1.06-1.10
	**NOC** (0)				
	1	3.89	1.23-12.33	0.95	0.38-2.36
	2	3.31	1.06-10.34	0.69	0.28-1.67
	3	4.32	1.39-13.41	0.82	0.34-1.96
	4 or 5	4.25	1.38-13.12	0.68	0.28-1.65
	6 or more	3.89	1.23-12.29	0.32	0.11-0.97
	**Diabetes** (Non-diabetes)				
	Diabetes	1.33	1.01-1.76	1.17	0.76-1.80
	**Hypertension Status** (Non-hypertensive)				
	Hypertensive	1.53	1.25-1.87	1.17	0.88-1.55
	**Occupation** (Employed)				
	Farmer	1.07	0.71-1.61	1.13	0.66-1.93

Jobless	1.00	0.69-1.45	0.91	0.44-1.90
Others	0.90	0.64-1.26	0.83	0.55-1.25
**Dietary salt intake** (Salt-light)				
Salt-medium(6-18g/day)	1.06	0.86-1.29	1.11	0.83-1.47
Salt-heavy(≥18g/day)	1.71	1.21-2.42	1.00	0.62-1.63
**Residence** (City)				
Rural	0.77	0.57-1.04	0.89	0.55-1.43
**Education** (Illiterate**)**				
Primary	1.02	0.79-1.33	1.31	0.88-1.94
Junior high school	0.60	0.39-0.92	1.08	0.66-1.76
Senior high school and	0.83	0.55-1.28	1.37	0.81-2.32
**Marital status** (In-marriage)				
Single	3.71	0.97-14.25	1.63	0.59-4.55
Divorced	0.73	0.23-2.39	0.69	0.16-2.94
Widowed	1.02	0.81-1.28	0.69	0.45-1.06
**Smoking history** (Never)				
Sometimes	0.65	0.38-1.13	0.85	0.57-1.27
Often	0.78	0.38-1.60	0.97	0.66-1.43
Quit	1.06	0.45-2.50	1.71	1.11-2.61
**Alcohol Drinking** (Never)				
Sometimes	1.00	0.68-1.46	1.14	0.83-1.57
Often	1.21	0.41-3.60	1.17	0.69-1.98
Quit	2.59	1.11-6.03	0.96	0.58-1.61

OR: odds ratio; CI: confidence interval
